# Heparanase Regulates Levels of Syndecan-1 in the Nucleus

**DOI:** 10.1371/journal.pone.0004947

**Published:** 2009-03-23

**Authors:** Ligong Chen, Ralph D. Sanderson

**Affiliations:** 1 Department of Pathology, University of Alabama at Birmingham, Birmingham, Alabama, United States of America; 2 Center for Metabolic Bone Disease and Comprehensive Cancer Center, University of Alabama at Birmingham, Birmingham, Alabama, United States of America; Johns Hopkins University, United States of America

## Abstract

Syndecan-1 is a transmembrane heparan sulfate-bearing proteoglycan known to regulate multiple biological functions at the cell surface and within the extracellular matrix. Its functional activity can be modulated by heparanase, an enzyme that cleaves heparan sulfate chains and whose expression has been associated with an aggressive phenotype in many cancers. In addition to remodeling syndecan-1 by cleaving its heparan sulfate chains, heparanase influences syndecan-1 location by upregulating expression of enzymes that accelerate its shedding from the cell surface. In the present study we discovered that heparanase also alters the level of nuclear syndecan-1. Upon upregulation of heparanase expression or following addition of recombinant heparanase to myeloma cells, the nuclear localization of syndecan-1 drops dramatically as revealed by confocal microscopy, western blotting and quantification by ELISA. This effect requires enzymatically active heparanase because cells expressing high levels of mutated, enzymatically inactive heparanase, failed to diminish syndecan-1 levels in the nucleus. Although heparan sulfate function within the nucleus is not well understood, there is emerging evidence that it may act to repress transcriptional activity. The resulting changes in gene expression facilitated by the loss of nuclear syndecan-1 could explain how heparanase enhances expression of MMP-9, VEGF, tissue factor and perhaps other effectors that condition the tumor microenvironment to promote an aggressive cancer phenotype.

## Introduction

Heparanase is an enzyme known to promote the progression of many cancers [1]. Its tumor promoting effects are due to enzymatic degradation of heparan sulfate that can, for example, liberate heparin-binding growth factors and remodel the extracellular matrix to facilitate tumor metastasis. In addition, via both enzymatic and non-enzymatic activities, heparanase can alter cell signaling with downstream effects on gene transcription [2]. Thus, heparanase is a multifunctional effector molecule whose complete repertoire of functions is still being elucidated.

It was recently discovered that heparanase can also enhance shedding of the syndecan-1 heparan sulfate proteoglycan from the surface of tumor cells [3], [4]. This occurs through heparanase-mediated upregulation of ERK phosphorylation leading to enhanced expression of MMP-9, a syndecan-1 sheddase [5]. The enhanced shedding of syndecan-1 is important biologically, because the shed proteoglycan remains active and can influence a wide array of behaviors such as tumor growth and metastasis, chemokine localization, leukocyte trafficking and pathogen virulence [6], [7], [8]. Thus, the change in location of syndecan-1 from the cell surface to the extracellular compartment has distinct and important pathological consequences.

Although heparan sulfate proteoglycans on the cell surface and within the extracellular matrix have been studied extensively, much less is known about their expression and function in the nucleus of cells. There are reports that syndecan-1 and other heparan sulfate proteoglycans are present in the nucleus [9], [10], [11], [12], [13]. The role of heparan sulfate in the nucleus has been linked to control of cell proliferation, shuttling of the heparin-binding growth factor FGF2, inhibition of DNA topoisomerase I activity and stabilization of the mitotic machinery [11], [12], [14], [15], [16]. The finding that heparan sulfate can inhibit DNA topoisomerase I activity suggests that its presence in the nucleus may inhibit gene transcription [15]. In addition, a recent study demonstrated that glycosaminoglycans, including heparin and heparan sulfate, can inhibit histone acetyltransferase (HAT) [17]. Because HAT facilitates transcriptional activation, these results also point to nuclear heparan sulfate as a repressor of gene transcription. This may be particularly important in pathological situations such as cancer, where abnormal HAT activity has been detected [18].

Given that heparanase promotes an aggressive tumor phenotype and that it can also regulate the location of syndecan-1, we examined the effect of heparanase expression on syndecan-1 localization within the nucleus. By confocal microscopy, western blotting and ELISA assay we demonstrate that when heparanase expression is increased in a human myeloma cell line, the level of syndecan-1 in the nucleus decreases dramatically. This ability of heparanase to regulate nuclear syndecan-1 may represent a mechanism whereby heparanase influences gene transcription with downstream effects that promote the aggressive tumor phenotype.

## Results

**Figure 1:**
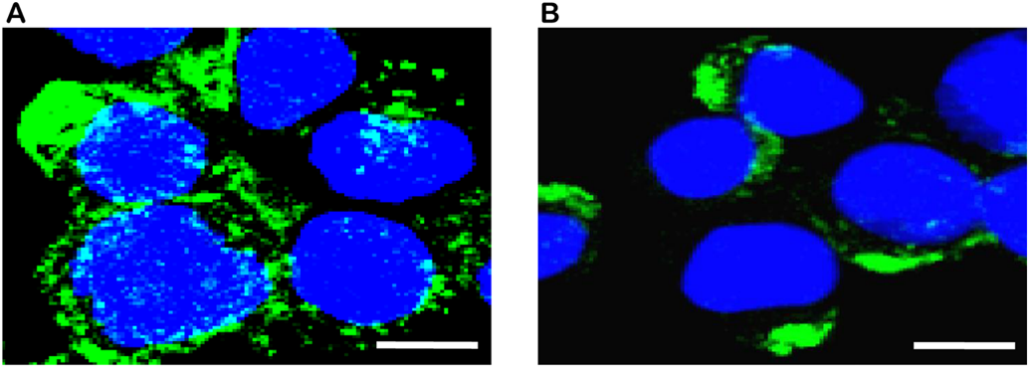
Syndecan-1 is not detected within the nucleus of cells expressing high levels of heparanase. Confocal microscopic z-stack images of (A) HPSE-low and (B) HPSE-high cells immunostained using antibody to syndecan-1. Blue (Hoechst stain) identifies nuclei; white identifies syndecan-1 within the nucleus (co-localization of Hoechst and syndecan-1); green identifies cytoplasmic and cell surface syndecan-1. Syndecan-1 is detected within nuclei of HPSE-low cells but absent in nuclei of the HPSE-high cells. Bar = 10 µm. Note: As is characteristic of myeloma cells, the size of the nucleus is large relative to the amount of cytoplasm.

Using confocal microscopy we noted that syndecan-1 was localized within the nucleus of CAG myeloma cells expressing low levels of heparanase (HPSE-low cells) but it not present within the nucleus of CAG cells expressing high levels of heparanase (HPSE-high cells) (Fig. 1). Cytoplasmic staining for syndecan-1 was present in both HPSE-low and HPSE-high cells but was more prominent in the HPSE-low cells. Both HPSE-low and HPSE-high cells exhibited bright staining on the cell surface, often in patches (Fig. 1). This cell surface pattern of staining is consistent with our previous finding that syndecan-1 is often found concentrated on uropods, structures present at the trailing edge of motile cells [19].

**Figure 2:**
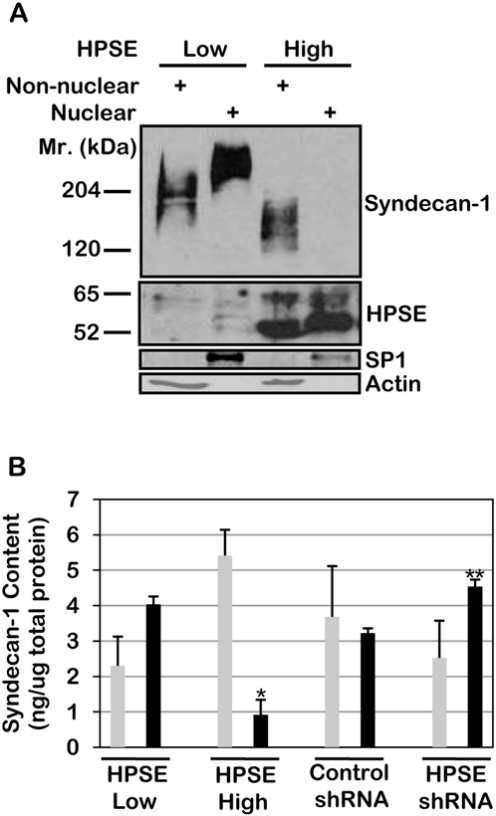
Elevated expression of heparanase dramatically decreases the level of syndecan-1 present within the nucleus. A) Nuclear and non-nuclear fractions were isolated from HPSE-low and HPSE-high cells (prepared using the pcDNA3 vector for transfections) and separated on SDS-PAGE. Western blots were probed with antibody to human syndecan-1, human heparanase, SP-1 or actin. B) Nuclear and non-nuclear fractions were isolated from HPSE-low and HPSE-high cells (prepared using the pIRES_2_ vector for transfections) and from wild-type CAG cells infected with control shRNA or an shRNA targeting heparanase. The quantity of syndecan-1 in each fraction was determined by ELISA. Grey bars = non-nuclear fraction; Black bars = nuclear fraction. Error bars represent standard error of the mean. *, *P*<0.01 vs. nuclear syndecan-1 in HPSE low cells; **, *P*<0.02 vs. nuclear syndecan-1 in shRNA control.

To examine further the nuclear localization of syndecan-1, cells were extracted and nuclear and non-nuclear fractions were isolated. (Although most studies refer to these as nuclear and cytoplasmic fractions, we use the terms nuclear and non-nuclear because the non-nuclear fraction includes not only cytoplasmic molecules but also cell surface molecules.) Western blots detected abundant syndecan-1 within the nuclear fraction of the HPSE-low cells but none within the nuclear fraction of the HPSE-high cells (Fig. 2A). Syndecan-1 is detected as a smear on western blots due to its heterogeneity in molecular size because of variations in glycosaminoglycan chain size and number [20]. As we have previously described, the size of syndecan-1 isolated from the HPSE-high cells is smaller than that from the HPSE-low cells due to heparanase cleavage of the heparan sulfate chains [21]. Immunostaining of the same blot with antibodies to heparanase revealed elevated levels of the 65 kDa (latent) and 52 kDa (active) enzyme were present in the nuclear and non-nuclear fractions of the HPSE-high cells as compared to HPSE-low cells. Detection of heparanase in the nucleus is consistent with previous reports [22]. Although not evident in this blot, our previous characterizations have revealed that low levels of heparanase are expressed by the HPSE-low cells [21]. Staining of the blot for SP1 and actin confirmed the fidelity of the nuclear and non-nuclear fractions, respectively (Fig. 2A). Interestingly, we found that SP1 levels were consistently lower in western blots of nuclear fractions of HPSE-high cells as compared to HPSE-low cells. This occurred even when the HPSE-high cells were expressing the mutated, enzymatically inactive form of the enzyme (data not shown). This raises the possibility that heparanase may be regulating levels of SP1 within the nucleus.

To ensure that the result above was not due to an artifact of the cell transfection process, we examined CAG cells that had been transfected with the cDNA for heparanase utilizing a different expression vector (pIRES_2_). In addition, as an alternative to assessing the fractions by Western blotting, the levels of syndecan-1 in the nuclear and non-nuclear fractions were quantified by ELISA. Results confirm the syndecan-1 levels in the nucleus drop dramatically when high levels of heparanase are expressed by these cells (Fig. 2B). In addition, shRNA knockdown of the endogenous expression of heparanase in wild-type CAG cells resulted in elevation of syndecan-1 levels in the nucleus (Fig. 2B). Not surprisingly, this increase in nuclear syndecan-1 was accompanied by a decrease in non-nuclear syndecan-1. This could be due to redistribution of non-nuclear syndecan-1 into the nucleus and/or due to an overall decrease in syndecan-1 expression upon heparanase knockdown [3]. Together these data reveal that the level of nuclear syndecan-1 in these cells correlates inversely with levels of heparanase expression.

**Figure 3:**
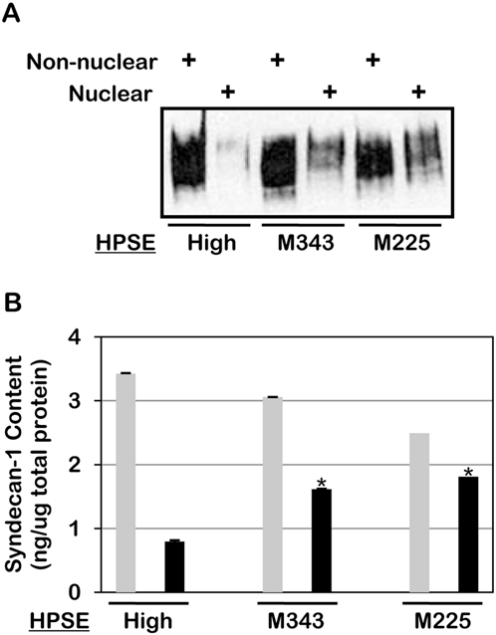
Heparanase enzymatic activity is required for reduction of syndecan-1 levels in the nucleus. Nuclear and non-nuclear fractions were prepared from CAG cells expressing high levels of wild-type heparanase (HPSE-high) or heparanase mutated at either amino acid 343 (M343) or amino acid 225 (M225) which renders them enzymatically inactive. All cells were prepared using pIRES_2_ vectors for transfections. Fractions were analyzed for syndecan-1 levels by A) western blotting and B) ELISA. Grey bars = non-nuclear fraction; Black bars = nuclear fraction. Error bars represent standard error of the mean. *, *P*<0.01 vs. nuclear syndecan-1 in HPSE high cells.

Because heparanase can have biological functions that are independent of its heparan sulfate degrading activity [1], we also examined nuclear syndecan-1 levels in cells transfected with mutated heparanase that lacks enzyme activity. Cells expressing these mutated forms of heparanase retained substantial nuclear syndecan-1 as compared to cells expressing the active enzyme (Fig. 3). This indicates that the regulation of syndecan-1 level in the nucleus is dependent, at least in part, on the heparan sulfate degrading activity of heparanase. In addition, cells transfected with the cDNA for the mutated enzyme act as an additional negative control for these studies because in each of the two mutation constructs only a single amino acid is altered, yet nuclear syndecan-1 levels are retained [3], [23].

**Figure 4:**
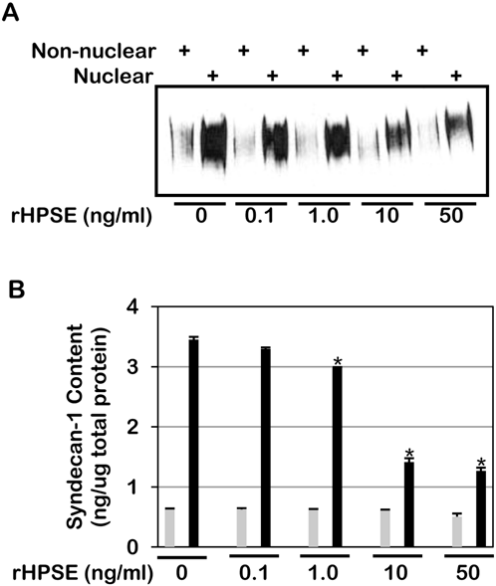
Exogenous recombinant heparanase (rHPSE) decreases nuclear syndecan-1 levels in a concentration-dependent manner. Recombinant heparanase was added to CAG cells having very low levels of heparanase expression (shRNA knockdown cells). Nuclear and non-nuclear fractions were prepared and syndecan-1 levels analyzed by A) western blotting and B) ELISA. Grey bars = non-nuclear fraction; Black bars = nuclear fraction. Error bars represent standard error of the mean. *, *P*<0.01 vs. nuclear syndecan-1 in cells treated with 0 ng/ml rHPSE.

Lastly, as a final confirmation of the effect of heparanase on nuclear syndecan-1 levels, exogenous heparanase was added to cells expressing very low levels of heparanase (heparanase knockdown cells). These experiments were feasible due to the ability of cells, including the CAG cells, to take up and utilize exogenously added heparanase [3], [24], [25]. In response to recombinant heparanase, the level of syndecan-1 present in the nucleus decreased in a concentration dependent fashion (Fig. 4). This confirms results obtained with heparanase transfected cells and strengthens the conclusion that heparanase regulates levels of syndecan-1 in the nucleus. Moreover, the finding that addition of exogenous heparanase can affect nuclear syndecan-1 levels indicates that the level of syndecan-1 in the nucleus of one cell can be altered by uptake of heparanase that was produced by another cell. Thus, heparanase may influence the nuclear localization of syndecan-1 broadly throughout the tumor microenvironment, even within cells lacking heparanase expression.

## Discussion

This work reveals that heparanase regulates the level of syndecan-1 present in the nucleus. This was demonstrated by showing that upregulation of heparanase caused decreased amounts of nuclear syndecan-1 using i) confocal microscopy, ii) Western blotting of nuclear and non-nuclear extracts, and iii) ELISA assays to quantify the amount of syndecan-1 present in nuclear and non-nuclear fractions. Upregulation of heparanase was achieved by either transfection with the cDNA for human heparanase into cells (using two different vectors) or by addition of exogenous heparanase to cells. In addition, cells expressing mutated heparanase lacking enzymatic activity retained high levels of nuclear syndecan-1 demonstrating that only the active form of the enzyme diminishes levels of nuclear syndecan-1.

Although heparan sulfate proteoglycans have been found within the nucleus, there are only limited reports regarding nuclear syndecan-1 [12], [16]. Syndecan-1 has not been previously reported to be localized in the nucleus of myeloma cells or in myeloma patient tumor cell samples. However, staining of bone marrow biopsies for syndecan-1 often includes counterstaining of nuclei which may prohibit detection of nuclear syndecan-1. In two reports, close scrutiny of biopsies stained with antibodies to syndecan-1 does reveal some nuclear staining [26], [27]. Also, syndecan-1 within the nucleus may not be assessable to antibody and thus not easily detected. This possibility is supported by our data in the present study where only moderate levels of syndecan-1 in the nucleus were detected in the heparanase-low cells by confocal microscopy, but in contrast, high levels of syndecan-1 were detected in the nuclear fraction of these cells by both western blotting and ELISA.

We do not yet know the mechanism whereby heparanase regulates the level of nuclear syndecan-1. Syndecan-1 lacks a nuclear localization signal [28] and thus may require a binding partner to shuttle it into the nucleus. Heparanase could influence the expression or function of this binding partner or, conversely, trimming of heparan sulfate chains by heparanase may render it no longer interactive with the shuttling molecule. Yet another possibility is that the onset of increased shedding of syndecan-1 caused by heparanase expression [3] results in more syndecan-1 being transported to the cell surface thereby making less available for nuclear transport. These possibilities await further investigation.

Heparanase expression has been linked to enhanced tumor aggressive behavior and poor prognosis in a number of cancers [1]. This role of heparanase is supported by findings that heparanase inhibitors can inhibit tumor growth and metastasis [29], [30], [31]. Heparanase can facilitate breakdown of the extracellular matrix which aids tumor cell migration and releases factors that promote tumor growth, angiogenesis and metastasis [1]. Additionally, there is growing evidence that heparanase can upregulate expression of genes that participate in creating aggressive behavior of tumors. These include VEGF, MMP-9, uPA/uPAR and tissue factor and likely others yet to be discovered [5], [32], [33]. Because at least some of these changes in gene expression occur downstream of signaling events (*e.g.*, ERK, Src), it is possible that these signals are responsible for loss of syndecan-1 nuclear localization and resulting upregulation of gene transcription.

Although heparan sulfates have been associated with several functional roles within the nucleus, its inhibition of gene transcription via inhibition of topoisomerase I and HAT activity are particularly intriguing [15], [17]. Regarding HATs, they regulate gene expression by catalyzing acetylation of the N-terminal region of histones, thereby modifying chromatin structure in a manner that facilitates transcriptional activation [18]. It was recently shown that both heparin and heparan sulfate can act as potent inhibitors of p300 and pCAF HAT activities [17]. Addition of heparin to pulmonary fibroblasts reduced histone H3 acetylation by 50% and Chinese hamster ovary cells deficient in glycosaminoglycan synthesis exhibited increased levels of acetylated histone H3 when compared to controls. The reduction in nuclear syndecan-1 that we detected following upregulation of heparanase expression thus could lead to increased histone acetylation with an associated increase in gene transcription.

We and others have speculated that heparanase acts as a master regulator of the aggressive tumor phenotype [2], [5]. This apparently is accomplished via heparanase effects on multiple cell behaviors including gene expression. Our findings indicate that heparanase regulation of gene expression may be related to its ability to inhibit accumulation of heparan sulfate proteoglycans within the nucleus. Thus, strategies to enhance nuclear heparan sulfate levels may prove effective in blocking at least some of the heparanase-mediated effects that promote tumor growth and metastasis.

## Materials and Methods

### Cells and transfections

CAG cells were isolated from a myeloma patient as previously described [19]. The cells were obtained and utilized following signed informed consent, in accordance with the Declaration of Helsinki. CAG cells with modified levels of heparanase expression have been previously extensively characterized [3], [5], [21] and include i) heparanase-low (HPSE-low) cells prepared by transfection with empty vector, ii) heparanase high (HPSE-high) cells prepared by transfection with vector containing the cDNA for human heparanase, iii) cells transduced with viral vectors containing a control shRNA sequence, and iv) cells transduced with viral vectors containing a shRNA sequence to knockdown heparanase expression.

### Treatment of cells with recombinant heparanase

Recombinant heparanase (kindly provided by Dr. Israel Vlodavsky and prepared as described [34]) at doses of 0.1, 1.0, 10 or 50 ng/ml was added to 6×10^6^ cells in 6 ml of complete RPMI medium, with a repeated addition of the same dose of heparanase 12 h later. 24 h after the initial dose of heparanase, cells and their conditioned medium were harvested. 1×10^6^ cells were used for analysis of cell surface expression of syndecan-1 by flow cytometry, and the remaining cells were used for preparation of nuclear and non-nuclear extracts.

### Preparation of nuclear and non-nuclear fractions

Nuclear and non-nuclear fractions were prepared as previously described [35]. 6×10^6^ cells were washed with cold PBS, centrifuged at 3000 RPM at 4°C for 5 minutes and cell pellets suspended in 0.5 ml of lysis buffer (10 mM Tris HCl, pH 7.4, 10 mM NaCl, 3 mM MgCl_2_, 0.5% NP40, 0.56 M Sucrose, and protease inhibitor cocktail). Cells were immediately dounced exactly 10 times in a small homogenizer (2 ml Kontes homogenizer, pestle overall×shaft O.D.(mm): 160×50, tube overall×reservoir O.D.(mm): 100×30). The lysate was transferred into a microcentrifuge tube, incubated on ice for 10 minutes and the cell lysate spun down at 3000 RPM at 4°C for 5 minutes. The supernatant represents the non-nuclear fraction and was transferred to another tube. The pellet was washed with 200 µl of hypotonic buffer (10 mM Hepes pH 7.9, 1.5 mM MgCl_2_, 10 mM KCl, and protease inhibitor cocktail) and centrifuged at 6000 RPM, 4°C for 5 minutes. The pellet was suspended in 100 µl of nuclear extraction buffer (20 mM Hepes pH 7.9, 20% glycerol, 600 mM KCl, 1.5 mM MgCl2, 0.2 mM EDTA, and protease inhibitor cocktail) and refrigerated at −80°C for 1 h. The extracts were thawed on ice, rotated at 4°C for 30 minutes and centrifuged at 14,000 RPM for 30 minutes. The supernatant (nuclear extract) was transferred to another tube, and stored at −80°C. For normalization, equal amounts of protein from the two fractions (as determined by BCA assay) were loaded for western blotting and ELISA.

### Western blots

For immunoblotting of syndecan-1, the extracts of nuclear and non-nuclear fractions were separated on 4% ∼15% gradient SDS-PAGE, transferred onto Nytran^+^ filters (Whatman/Schleicher&Schuell, Florham Park, NJ), probed with antibody B-A38 (1:000; Cell Sciences, Inc., Norwood, MA) followed by a horseradish peroxidase–conjugated secondary anti-mouse antibody (1:3000; GE Healthcare, Pittsburgh, PA), and visualized by chemiluminescence (GE Healthcare, Pittsburgh, PA). For immunoblotting of heparanase, SP1 or actin, the cell extracts were separated on 10% SDS-PAGE, transferred onto nitrocellulose filters (Whatman/Schleicher&Schuell), probed with anti-heparanase (1:000; [21]), anti-SP1 (1:1000, Santa Cruz Biotechnology, Santa Cruz, CA) or anti-actin (1:500, Santa Cruz Biotechnology) antibodies, followed by a horseradish peroxidase–conjugated secondary antibody (1:3000; GE Healthcare), and visualized by chemiluminescence. For western blots, data shown are representative blots from a minimum of three independent experiments.

### Enzyme-linked immunosorbent assay (ELISA)

The levels of syndecan-1 present in 2.5 µg of protein from each fraction were measured by an Eli-pair kit from Diaclone (Cell Sciences, Inc.). The syndecan-1 content was calculated as the number of nanograms of syndecan-1 in each microgram of total protein in cell extracts or the number of nanograms of syndecan-1 in each milliliter of conditioned medium. The ELISA data shown are from two separate experiments with duplicate wells for each experimental point. Thus, each bar represents four independent determinations.

### Immunocytochemistry

Cells were fixed with 3% formaldehyde in PBS for 45 min at room temperature. 70 ul of cell suspension was loaded into the cytospin funnel and spun onto a slide at 1000 RPM for 5 min. The cells on the slides were postfixed with 3% formaldehyde in PBS for 5 min. Cells were permeablized with 0.5% Triton X-100 in PBS for 3 min at room temperature and rinsed in PBS with 3 quick changes. The slides were incubated with 1% BSA in PBS for 30 min at room temperature followed by incubation with FITC-conjugated or unconjugated B-A38 (1:20 in 1% BSA in PBS) at 4°C overnight followed by room temperature for 2 h. After washing, the cells incubated with unconjugated B-A38 were further incubated with antimouse IgG-Alexa 594 (1:100; Invitrogen, Carlsbad, CA) in 1% BSA in PBS for at room temperature 1 h. After washing in PBS, cells were stained with Hoechst 33258 (Invitrogen) at 20 µg/ml in PBS for 4 min to label nuclei. Cells were viewed and photographed using confocal laser microscopy.
